# Nicotinamide Treatment Facilitates Mitochondrial Fission through Drp1 Activation Mediated by SIRT1-Induced Changes in Cellular Levels of cAMP and Ca^2+^

**DOI:** 10.3390/cells10030612

**Published:** 2021-03-10

**Authors:** Seon Beom Song, Jin Sung Park, So Young Jang, Eun Seong Hwang

**Affiliations:** Department of Life Science, University of Seoul, Jeonnong-dong, Dongdaemun-gu, Seoul 02504, Korea; scitiger@naver.com (S.B.S.); kaiba_man@naver.com (J.S.P.); syjang@medytox.com (S.Y.J.)

**Keywords:** nicotinamide, mitochondrial fragmentation, SIRT1, AMPK, Drp1, calcineurin

## Abstract

Mitochondrial autophagy (or mitophagy) is essential for mitochondrial quality control, which is critical for cellular and organismal health by attenuating reactive oxygen species generation and maintaining bioenergy homeostasis. Previously, we showed that mitophagy is activated in human cells through SIRT1 activation upon treatment of nicotinamide (NAM). Further, mitochondria are maintained as short fragments in the treated cells. In the current study, molecular pathways for NAM-induced mitochondrial fragmentation were sought. NAM treatment induced mitochondrial fission, at least in part by activating dynamin-1-like protein (Drp1), and this was through attenuation of the inhibitory phosphorylation at serine 637 (S637) of Drp1. This Drp1 hypo-phosphorylation was attributed to SIRT1-mediated activation of AMP-activated protein kinase (AMPK), which in turn induced a decrease in cellular levels of cyclic AMP (cAMP) and protein kinase A (PKA) activity, a kinase targeting S637 of Drp1. Furthermore, in NAM-treated cells, cytosolic Ca^2+^ was highly maintained; and, as a consequence, activity of calcineurin, a Drp1-dephosphorylating phosphatase, is expected to be elevated. These results suggest that NAD^+^-mediated SIRT1 activation facilitates mitochondrial fission through activation of Drp1 by suppressing its phosphorylation and accelerating its dephosphorylation. Additionally, it is suggested that there is a cycle of mitochondrial fragmentation and cytosolic Ca^2+^-mediated Drp1 dephosphorylation that may drive sustained mitochondrial fragmentation.

## 1. Introduction

Mitochondrial quality, which is facilitated by coordination of degradation of defective mitochondria and their replacement by biogenesis, is one critical factor in keeping the levels of reactive oxygen species (ROS) low; therefore, it plays key roles for health and longevity of cells as well as tissues [1]. However, under oxidative stress or during senescence or aging of cells, mitochondrial autophagy (mitophagy), a major mechanism for the removal of damaged mitochondria, is frequently impaired, and therefore mitochondrial quality deteriorates [2,3,4,5,6,7].

Mitochondria exist as tubular networks in normal cells, and for mitophagic removal of damaged mitochondria, their sorting and fragmentation are important prerequisites. This structural dynamics of mitochondria is mediated by fusion factors such as mitofusins (Mfn) I and II [8] and fission factors such as mitochondrial fission 1 protein (Fis I) and dynamin-1-like protein (DrpI) [9,10]. However, this is downregulated during aging and senescence [11,12], and the number of diseases and conditions that are linked with defects in mitochondrial dynamics, which include heart failure and Parkinson’s disease, is increasing rapidly [13,14,15]. Therefore, improved understanding of the details of mitochondrial dynamics and their coordination to autophagy along with the development of the tools that would allow mitochondria modulation would greatly facilitate prevention as well as treatment of these aging-related diseases.

Nicotinamide (NAM), a form of vitamin B3 and a precursor of NAD^+^, has been shown to promote survival and longevity of a variety of cell types [16,17,18]. A long-term treatment of NAM at 5 mM substantially delayed the onset of senescence in normal human fibroblasts, keratinocytes, and bone marrow stem cells [16,18]. The treatment decreased mitochondrial content and superoxide generation, and it also caused an increase in mitochondrial quality. This was found to be driven mainly through autophagy activation mediated by SIRT1 [6,19], which deacetylates and activates a number of autophagy gene proteins such as ATG7 and ATG8 [20,21]. On the basis of these findings, we hypothesized that NAM treatment facilitates mitochondrial quality enhancement and, thereby, lowers oxidative stress allowing cellular longevity. Another remarkable change in the NAM-treated cells is prolonged maintenance of mitochondrial fragmentation [6], a change that is expected to facilitate mitophagy. Meanwhile, this sustained mitochondrial fragmentation suggests that fission might be ongoing in the treated cells even after mitophagy has reached a steady state. Further understanding of these changes may provide valuable information on the physiological modulation of mitochondrial fission and its coordination with autophagy initiation and may provide strategies for mitochondrial quality enhancement.

Mitochondrial fission is executed by Drp1, multiples of which form a ring of oligomers and divide mitochondria [22]. Drp1 activity is regulated by multiple post-translational modifications, among which phosphorylation has been most studied. Drp1 phosphorylation at serine 616 (S616) by Cdk1/cyclin B is known to cause mitochondrial fragmentation upon cell cycle progression [23]. On the other hand, Drp1 phosphorylation at S637 by the cyclic AMP (cAMP)-dependent protein kinase A (PKA) inhibits mitochondrial fission through preventing translocation to mitochondria [24,25,26]. Additionally, dephosphorylation at S637 by a Ca^2+^-dependent phosphatase calcineurin increases Drp1 recruitment to mitochondria and thereby promotes mitochondrial fission [26]. These and our previous findings indicate a tight link of mitochondrial structural dynamics to cellular physiology and signaling as well as energy status.

In this study, we found that Drp1 becomes hypo-phosphorylated and activated upon NAM treatment, and we investigated the molecular mechanisms underlying this change. The study found activation of the self-stimulatory cycle for accelerating and sustaining mitochondrial fission by elevation of the NAD^+^ level. Our results suggest the importance of keeping high levels of NAD^+^ for mitochondria quality maintenance.

## 2. Materials and Methods

### 2.1. Cell Culture and Chemicals

Normal human fibroblasts were expanded from primary explant isolated from healthy newborn foreskin and provided by Dr. Joon Ho Jung (Seoul National University, Korea) (IRB No. H-1101-116-353 of the School of Medicine, Seoul National University). NIH-H460 and MCF7 cell lines were purchased from American Type Cell Culture (Manassas, VA 20110, USA). The cells were cultured in Dulbeco’s modified Eagle’s media (DMEM) plus 10% fetal bovine serum (FBS) supplemented with or without 5 mM NAM (or NAD^+^). To the culture media, the following chemicals were added: FK866 (SC-205325), compound C (SC-200689), and 8-br-cAMP (SC-201564) from Santa Cruz Biotechnology (Dallas, TX, USA); SRT1720 (567860), EX527 (E7034), sirtinol (S7942), forskolin (F6886), ionomycin (I9657), and FK506 (F4679) from Sigma-Aldrich Co. (St. Louis, MO, USA); Aicar (2626926) and IBMX (2885842) from Peprotech (Seoul, Korea); and Fluo-3 AM (F1241) and Fluo-4 AM (F14201) from Thermo Fisher (Waltham, MA, USA).

### 2.2. Western Blot Analysis

Cells were lysed with RIPA buffer (50 mM Tris-HCl [pH 7.5], 150 mM NaCl, 1% Nonidet P-40, 0.5% sodium deoxycholate, 0.1% SDS) supplemented with NaF, NaVO_4_, and a protease inhibitor mixture (Sigma-Aldrich). Typically, 20–30 μg of protein was separated by SDS-PAGE and transferred to a nitrocellulose membrane. Protein bands were visualized by blotting with antibodies to follow human proteins and horseradish peroxidase-conjugated secondary antibodies and reaction with SuperSignal WestFemto substrate (Thermo Fisher). Phosphor-Drp1 (S637) (4867S), phosphor-Drp1 (S616) (3455S), phosphor-AMPKα (T172) (2535S), AMPKα (2603S), acetyl-p53 (K382) (2525S), and SIRT1 (8469) were obtained from Cell Signaling Technology (Beverly, MA, USA); Drp1 (611112) and Opa1 (612606) from BD Biosciences (Franklin Lakes, NJ, USA); p53 (DO-1), Mfn1 (SC-166644), Tom20 (SC-17764), and ERK1/2 (SC-93) from Santa Cruz Biotechnology; β-actin (A5441) from Sigma-Aldrich; and calcineurin (PA5-17446) from Thermo Fisher.

### 2.3. Flow Cytometry for Determination of Cellular Mitochondrial Content

Cells were treated with 5 mM NAM for 72 h and stained with 30 nM MitoTrackerGreen (M7514, Thermo Fisher) at 37 °C for 30 min. After washing in phosphate-buffered saline (PBS), cells were analyzed by flow cytometry on cellular mitochondrial content using FACS Canto II (BD Biosciences).

### 2.4. Confocal Microscopy and Immunofluorescence

To visualize mitochondria, cells cultured on a cover slip were immune-stained with antibodies against OXPHOS (MS601, Mitosciences, Eugene, OR, USA) and Tom20 (SC-11415, Santa Cruz Biotechnology). In brief, cells were fixed in 3.7% paraformaldehyde in PBS for 20 min, permeabilized with 0.1% Triton X-100 for 15 min, blocked with 10% FBS in PBS for 2 h, and incubated with antibodies. To observe Drp1 proteins, after staining with Drp1 antibody (611112, BD Biosciences), cover slips were applied to confocal microscopy (LSM 510, Carl Zeiss) and photographed. Mitochondrial length was measured using ImageJ analysis software (v1.52e, National Institutes of Health, Bethesda, MD, USA). To visualize Ca^2+^ level in cells, cells were cultured in a glass-bottom dish and stained with 1 µM Fluo-3 AM (F1241) or Fluo-4 AM (F14201) from Thermo Fisher for 1 h at 37 °C. After staining, cells were washed with PBS and incubated in media for a further 20 min to allow complete de-esterification of intracellular AM esters. Samples were mounted and visualized with a fluorescence microscope (DE/Axio imager A1, Carl Zeiss).

### 2.5. Transfection with siRNA

Cells seeded on a chamber slide were transfected with either negative control RNA (siNeg), siRNA against human SIRT1 (siSIRT1; CUUGUACGACGAAGACGAC), AMPK (siAMPK; ATGATGTCAGATGGTGAATTT), Drp1 (siDrp1; GCAGAAGAAUGGGGU AAAU), Fis1 (siFis1; CGAGCUGGUGUCUGUGGAG), Mfn1 (siMfn1; pre-designed, 55669), or calcineurin (siPPP3CA; pre-designed, 5530) (all from Bioneer, Daejun, Korea) by using Lipofectamine RNAiMAX (13778-150, Thermo Fisher) according to the manufacturer’s protocol. Cells were incubated for 2–3 days before analysis. The siNeg RNA (CCUACGCCACCAAUUUCGU (dTdT)) has low homology to any human gene.

### 2.6. Measurement of Cellular cAMP Level

Equal numbers of cells (typically in the range of 1–5 × 10⁵) were lysed to determine the level of cellular cAMP by using a cAMP assay kit (4339S, Cell Signaling Technology) according to the manufacturer’s protocol.

### 2.7. Determination of Cytosolic Ca^2+^ Level

To measure the cytosolic calcium level, cells were treated with 5 mM NAM or 1 µM SRT1720 for an indicated time period and stained with 1 µM Fluo-4 AM at 37 °C for 1 h. After washing in PBS, cells were incubated in media for a further 20 min to allow complete de-esterification of intracellular AM esters and then analyzed by flow cytometry using a FACS Canto II (BD Biosciences). For a positive control, 2 µM ionomycin was used.

### 2.8. Statistical Analysis

For all quantitative presentation of protein band intensities, measurements were made in triplicate, and mean ± standard deviation (S.D.) is presented. Homogeneity of variances was assessed by a Bartlett test. For mitochondrial length and cellular fluorescence intensity, measurements were made for at least 20 cells and mean ± S.D. is presented. Intergroup comparison of the mean values was performed by one-way analysis of ANOVA using InStat 3.06 (GraphPad Software Inc., San Diego, CA, USA). A *p*-value less than 0.05 is considered to be statistically significant.

## 3. Results

### 3.1. Drp1 Is Activated and Induces Mitochondrial Fragmentation upon NAM Treatment

**Figure 1:**
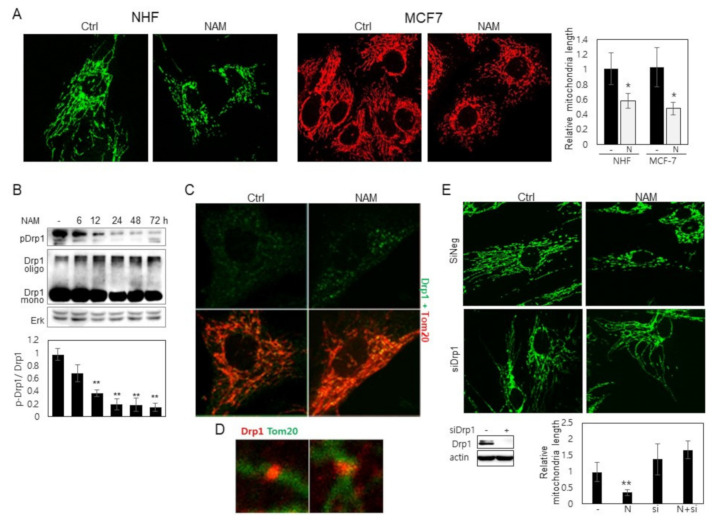
NAM induces mitochondrial fragmentation and Drp1 activation. (**A**) Human fibroblasts and MCF-7 cells were cultured in the presence of 5 mM NAM for 48 h. After fixing the cells, mitochondria were labeled with antibodies to OXPHOS (NHF) or Tom20 (MCF-7) and visualized in confocal microscopy. Length of mitochondria in 20 different cells was measured and averaged, and the mean value relative to that in untreated cells is presented as a bar in the graph at right (*, *p* < 0.05 vs. (-)). (**B**) Fibroblasts cultured in the presence of 5 mM NAM for indicated periods were applied to western blotting against phosphor-Drp1 (S637) or Drp1. Protein bands were quantitated by densitometry. Mean values of the measurement of the bands from three independent experiments are presented in a bar graph below (**, *p* < 0.01 vs. (-)). (**C**) Fibroblasts were incubated for 12 h on cover slips in the absence or presence of 5 mM NAM. The cells were then immuno-stained by Tom20 and Drp1 antibodies and applied to confocal imaging for mitochondria (red) and Drp1 protein (green). In (**D**), two enlarged images are presented to show Drp1 puncta (red) localized on a mitochondrial thread (green). (**E**) Cells transfected with siNeg RNA or siDrp1 RNA were cultured in the presence of 5 mM NAM for 48 h. After fixing, mitochondria were immune-stained with OXPHOS antibody and visualized. Length of mitochondria in at 10 different cells was measured and averaged, and the mean value relative to that in untreated cells is presented as a bar in the graph at bottom. (*, *p* < 0.05 vs. (-)).

NAM treatment activated autophagy in tested human cells including normal fibroblasts and cancer-derived cells [6,19]. It also induced mitochondrial fragmentation, and thereby facilitated mitophagy, which drives a decrease in cellular mitochondrial mass of up to 30% in 2–3 days in tested cells (Supplemental Figure S1A). Accordingly, the average length of mitochondria decreased gradually, and many of them, especially at the cell periphery, appear as short dots as presented in human fibroblasts, MCF7, and H460 cells (Figure 1A, and Supplemental Figure S1B). This fragmental presence of mitochondria can be attributed to either poor fusion or accelerated fission. The possibility of the latter was investigated with a focus on the involvement of Drp1 activation, because Drp1 knockdown abolished NAM-induced decrease of mitochondrial content, whereas knocking down Fis1 or Mfn1 mRNA did not affect the effect of NAM (Supplemental Figure S1C). Although these brief examinations do not rule out any involvement of these or any other factors, we decided to investigate Drp1 first because of the relative abundance of molecular mechanisms underlying Drp1 activity regulation. Drp1 activity is negatively modulated mainly by phosphorylation at S637, a target of various factors including PKA [27]. Whether phosphorylation status at this site is altered upon NAM treatment was investigated. Western blotting shows a decrease in the level of phosphor-Drp1 (at S637), which is apparent from as early as 12 h of NAM treatment (Figure 1B). Dephosphorylation at this site has been reported to promote Drp1 recruitment to mitochondria. Meanwhile, Drp1 phosphorylation at S616 was not found to be affected by NAM treatment at least for 48 h (Supplemental Figure S2). It is also known that active Drp1 molecules aggregate and localize to mitochondria [28]. Confocal imaging supports Drp1 activation. Upon NAM treatment for 12 h, large Drp1 puncta were formed, and many of them co-localized with mitochondrial filaments (Figure 1C, green in upper panel and yellow puncta in lower panel) (these structures appeared as early as from 2 h of NAM treatment.) Additionally, in magnified confocal images, some Drp1 proteins appeared to be present in a band wrapping the wrist of lengthy mitochondria (Figure 1D). Furthermore, knocking down Drp1 expression by using siRNA abolished the NAM-induced mitochondrial fragmentation (Figure 1E (NAM versus NAM + siDrp1)). Together, these results demonstrate that NAM treatment induces Drp1 activation through hypo-phosphorylation at S637.

### 3.2. NAM-Induced Mitochondrial Fragmentation Is Mediated by SIRT1 Activation

**Figure 2:**
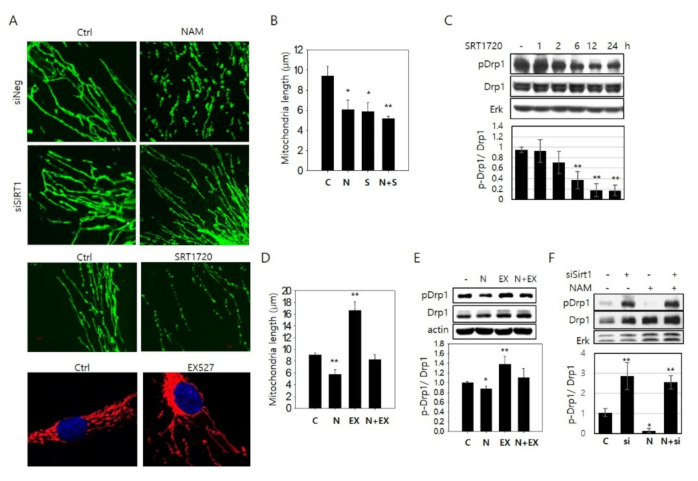
NAM-induced mitochondrial fragmentation and Drp1 hypo-phosphorylation are mediated through SIRT1 activation. (**A**,**B**) Fibroblasts were transfected with siNeg or siSIRT1 RNA. After 48 h, the cells were incubated with 5 mM NAM for 48 h (upper panels). Fibroblasts were treated with 1 μM SRT1720 for 24 h or EX527 for 12 h (lower panels). After the treatments, mitochondria were immuno-stained with OXPHOS antibody and visualized in confocal microscopy (**A**), and mitochondrial length in at least 10 cells was quantified by ImageJ analysis and graphed (**B**). (**C**) Cells treated with 1 μM SRT1720 for indicated periods were lysed and applied to immunoblot analysis for phosphor-Drp1 or Drp1. (**D**) Cells were incubated on cover slips with 5 mM NAM for 48 h and treated with 1 μM EX527 for the last 12 h of the total period. Mitochondrial length in the confocal microphotographs was determined as in (**A**). (**E**) Cells were treated as in (**D**), and phosphor-Drp1 and Drp1 protein bands were detected by immunoblot analysis. (**F**) Cells transfected with either siNeg RNA or siSIRT1 RNA were incubated in the absence or presence of 5 mM NAM for 48 h. Then, cells were lysed and applied to immunoblot analysis with antibodies against phosphor-Drp1 or Drp1. For (**C**,**E**,**F**), protein bands were quantitated using a densitometer, and relative intensities (average of three biological repeats) were plotted in bar graphs (*, *p* < 0.05 and **, *p* < 0.01 vs. (-)).

Our previous study showed that treatment of NAD^+^ also induces mitochondrial fragmentation ([19] and Supplemental Figure S3A). SIRT1 activation upon NAM treatment and the involvement of its activation in mitochondrial fragmentation were also demonstrated in our previous study ([19] and also shown in Supplemental Figure S3B). These are confirmed in the current study. NAM-induced mitochondrial fragmentation is dependent on the expression of SIRT1, and SIRT1 activation by the treatment of SRT1720, a potent SIRT1 activator [29], induces mitochondrial fragmentation, while the treatment of an inhibitor, EX527, causes their lengthening (Figure 2A,B,D), which together indicate that SIRT1 activated through the elevation of NAD^+^ drives mitochondrial fission in NAM-treated cells. Confirming this, SIRT1 activation induced gradual hypo-phosphorylation of Drp1 (Figure 2C). Also observed was the failure of mitochondrial fragmentation by NAM upon co-treatment with EX527, a potent SIRT1 inhibitor, which by itself caused an increase in mean length of mitochondria (Figure 2D) and the phosphorylation level of Drp1 in control as well as NAM-treated cells (Figure 2E). Treatment of siSIRT1 RNA or another SIRT1 inhibitor (sirtinol) also caused an increase in Drp1 phosphorylation (Figure 2F and Supplemental Figures S3C and S4). Overall, these results indicate that SIRT1 induces mitochondrial fragmentation by driving Drp1 hypo-phosphorylation. Finally, the increase in Drp1 phosphorylation upon treatment of FK866, an inhibitor of nicotinamide phosphoribosyltransferase (NAMPT) [30], which facilitates conversion of NAM to NAD^+^ [31] (Supplemental Figure 3C), also demonstrates that Drp1 hypo-phosphorylation and mitochondrial fragmentation upon NAM treatment are an event mediated by an increase of SIRT1 activity through an increase in NAD^+^ availability.

### 3.3. SIRT1-Mediated AMPK Activation Drives Drp1 Hypo-Phosphorylation

**Figure 3:**
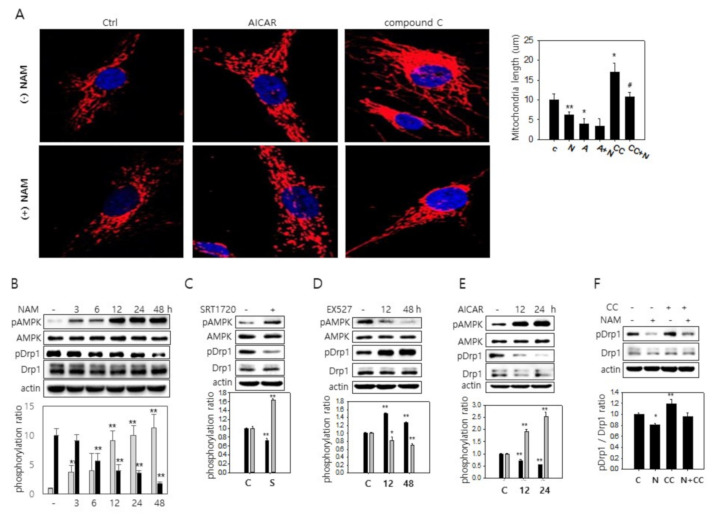
Direct activation of AMPK decreases phosphorylation of Drp1. (**A**) Fibroblasts on cover slips were either mock-treated or treated with 5 mM NAM for 48 h. During incubation, cells were further treated with 500 nM AICAR for the last 24 h or 1 μM compound C for the last 3 h. Cells were then fixed, and mitochondria (red) were visualized by confocal microscopy using Tom20 antibody. Length of mitochondria in at least 20 cells from two different slides was measured and averaged, and plotted in the bar graph. Nuclei were counter-stained with DAPI (blue). (*, *p* < 0.05 and **, *p* < 0.01 vs. control. #, *p* < 0.05 between CC and CC + N). (**B**–**F**) Cells were cultivated with 5 mM NAM (for the indicated period), 1 µM SRT1720 (for 24 h), 1 µM EX527, 500 nM AICAR (for indicated time period), or 1 µM compound C (for 3 h). Protein extracts were analyzed by immunoblot analysis with antibodies against phosphor-AMPK, AMPK, phosphor-Drp1, Drp1, and β-actin. Protein bands were quantitated through densitometry, and the measured values from three-independent western blots were averaged. Relative values of phosphor-AMPK over AMPK (grey bars) and phosphor-Drp1 over Drp1 (black bars) were normalized by the values of control cells (-), and the relative values were plotted and presented in bar graphs. (*, *p* < 0.05 and **, *p* < 0.01 vs. control).

Next, we investigated how SIRT1 activation drives Drp1 activation. SIRT1 activates AMPK via deacetylation and activation of liver kinase B1 (LKB1), an upstream kinase of AMPK [32,33]. The involvement of AMPK activity in mitochondrial fission has also been suggested [34]. Indeed, treatment of AMPK activator, 5-aminoimidazole-4-carboxamide 1-β-D-ribofuranoside (AICAR), caused mitochondria shortening (Figure 3A). In contrast, treatment of compound C, a potent AMPK inhibitor, caused an increase in mitochondria length in both control and NAM-treated cells (Figure 3A). Because SIRT1 activity appears to work at least partially through Drp1 hypo-phosphorylation, we decided to determine whether AMPK activity affects Drp1 phosphorylation status. In a previous study, mitochondrial fission factor (MFF), a mitochondrial receptor for Drp1 [35,36], was suggested as a target of AMPK activity [34]. However, the involvement of Drp1 in AMPK-mediated mitochondrial fission has not been investigated. Treatment of NAM induced gradual activation of AMPK, and this matched well with the changes in the level of phosphor-Drp1 (Figure 3B). Similarly, treatment of SRT1720 caused an increase in phosphor-AMPK and a decrease in phosphor-Drp1 (Figure 3C). In the same vein, a decrease in AMPK activity upon treatment of EX527 or siSIRT1 RNA contrasted with Drp1 hyper-phosphorylation (Figure 3D and Supplemental Figure S4B,C). Additionally, treatment of AICAR caused Drp1 hypo-phosphorylation in inverse correlation to phosphorylation of AMPK (Figure 3F), whereas treatment of compound C or siAMPK RNA attenuated NAM-induced decrease in Drp1 phosphorylation as well as mitochondrial fragmentation (Figure 3F and Supplemental Figure S5). These results together suggest the possible involvement of AMPK activation in NAM-induced Drp1 activation and mitochondrial fission.

### 3.4. Possible Downregulation of PKA Activity and Drp1 Hypo-Phosphorylation through a Decrease in cAMP Level in NAM-Treated Cells

**Figure 4:**
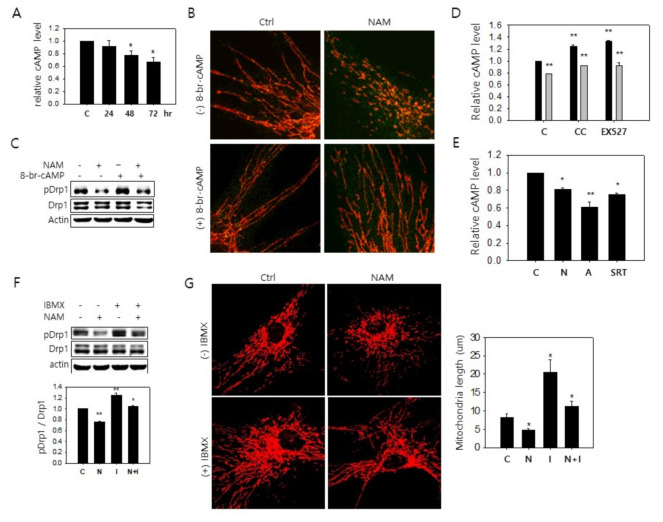
NAM-induced Drp1 hypo-phosphorylation is driven by a decrease of cAMP. (**A**) Human fibroblasts cultivated with 5 mM NAM for 24, 48, or 72 h were lysed and applied to measurement of cellular cAMP level. Amounts of cAMP per cell were normalized by that of untreated control cells, and the relative values were plotted. (**B**,**C**) Cells were incubated with 5 mM NAM for 48 h with addition of 100 µM 8-br-cAMP for the last 1 h, and then fixed and stained with antibodies against Tom20 (red) or Drp1 (green) and photographed by confocal microscopy (**B**), or lysed and applied to western blotting for phosphor-Drp1 (**C**). (**D**,**E**) Cells were either mock-treated (black bars) or treated with 5 mM NAM (grey bars) for 72 h with addition of 1 µM compound C (CC), 1 μM EX527, 500 nM AICAR (**A**), or 1 µM SRT1720 for the last 3, 24, 24, or 24 h, respectively. Cells were lysed and cellular cAMP levels were quantitated. Values from three independent measurements were averaged, normalized by the values of the control cells, and plotted. (**F**) Cells were incubated with 5 mM NAM for 48 h with addition of 500 nM IBMX for the last 1 h, lysed, and applied to immunoblot analysis with antibodies against phospho-Drp1 and Drp1. Mean densitometric quantity of protein bands in three independent western blots was normalized by that of untreated cells, and plotted (*, *p* < 0.05 and **, *p* < 0.01 vs. control). (**G**) Cells were incubated with 5 mM NAM for 48 h with addition of 500 nM IBMX for the last 1 h. Cells were fixed and mitochondria (red) were visualized with confocal microscopy using Tom20 antibody. Average mitochondria length was measured using at least 10 cells and values relative to those of untreated control cells were plotted in the graph (*, *p* < 0.05 vs. control).

A study was carried out to discover how AMPK activation is linked to Drp1 hypo-phosphorylation. First, any alteration of PKA activity was investigated by examining the cellular levels of cAMP, a key factor for PKA activation. Upon NAM treatment, cAMP gradually decreased to reach levels below the 70% level of the untreated cells in three days of NAM treatment (Figure 4A). Meanwhile, treatment of 8-bromo-cAMP, a membrane-permeable analogue of cAMP, attenuated NAM-induced mitochondrial fragmentation, thereby confirming PKA modulation of Drp1 activity (Figure 4B) and its direct Drp1 phosphorylation (Figure 4C, lanes 2 vs. 4). These results also suggest that NAM induces mitochondrial fragmentation at least partially through modulating the level of cAMP. The cellular level of cAMP and PKA activity can be subject to modulation by AMPK, because AMPK phosphorylates and activates phosphodiesterase 4B (PDE4B), which converts cAMP to AMP and thereby causes a reduction in the levels of PKA activity [37]. The inhibition of AMPK by compound C increased the cAMP level in control cells and also attenuated the decrease of the cAMP level in NAM-treated cells (Figure 4D). Treatment of EX527 resulted in similar effects (Figure 4D). A combined treatment of compound C and EX527 did not show a significant additive effect (data not shown). These results link SIRT1 and AMPK to the cAMP level as a mechanism underlying Drp1 hypo-phosphorylation upon NAM treatment. Similarly, the level of cAMP decreased by the treatment of the activators of these proteins, SRT1720 and AICAR (Figure 4E). The role of cAMP in the alteration of Drp1 phosphorylation was further supported by experiments determining the effect of isobutylmethylxanthine (IBMX), which increases cAMP by inhibiting phosphodiesterases and stimulating adenylate cyclase [38,39]. IBMX treatment attenuated the decrease of the levels of Drp1 phosphorylation and cAMP, the events induced by NAM treatment (Figure 4F and Supplemental Figure S6A). IBMX treatment also caused lengthening of mitochondria, and this was attenuated by NAM treatment (Figure 4G). NAM treatment was also shown to attenuate the increase of Drp1 phosphorylation (Supplemental Figure S6B,C) upon the treatment of forskolin, an activator of cAMP-converting enzyme, adenylyl cyclase [Forskolin, Drugs.com, retrieved on March 18, 2020]. Together, these results suggest that AMPK activation decreases the cAMP level and PKA activity and thereby may drive Drp1 hypo-phosphorylation in NAM-treated cells.

### 3.5. Increase in Cytosolic Ca^2+^ and Calcineurin Activation May Also Be Involved in Drp1 Hypo-Phsophorylation in NAM-Treated Cells

Treatment of EX527, sirtinol, compound C, 8-br-cAMP, or IBMX attenuated the effect of NAM on Drp1 hypo-phosphorylation, but it did not completely abolish it (Figure 2B, Figure 3B, Figure 4B,F). Furthermore, the rapid decrease of Drp1 phosphorylation levels (Figure 1C,G, and Figure 2C) contrasted with a rather slow decrease of cAMP levels (Figure 4A). This may suggest the possibility of additional routes in NAM-driven Drp1 hypo-phosphorylation.

**Figure 5:**
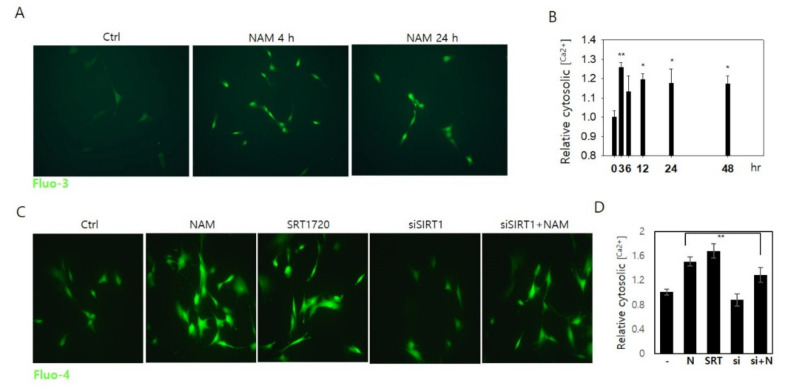
SIRT1 activation by NAM treatment increases cellular Ca^2+^. (**A**) To determine changes in cytosolic calcium level upon 5 mM NAM treatment, fibroblasts were stained with Fluo-3 at 4 or 24 h during incubation and visualized in a fluorescence microscope. (**B**) Fibroblasts incubated in the presence of 5 mM NAM were stained with Fluo-4 at indicated time points and applied to flow cytometry for quantification of cellular calcium levels. (*, *p* < 0.05 and **, *p* < 0.01 vs control (0)) (**C**) Normally, growing cells or those transfected with siSIRT1 RNA were mock-treated or treated with 5 mM NAM or 1 μM SRT1720 for 24 h, and stained with Fluo-4 and observed by fluorescence microscopy. (**D**) Cells treated as in (**C**) were applied to flow cytometry for Fluo-4 fluorescence. Mean values of cells from three independent cultures were normalized by those of untreated control cells (**, *p* < 0.01 between NAM treatments in normal vs. siSIRT1-transfected cells).

Drp1 is dephosphorylated by calcineurin [25], a Ca^2+^-dependent phosphatase. Possible involvement of calcineurin activation in Drp1 hypo-phosphorylation in NAM-treated cells is suggested by an increase of cytosolic Ca^2+^ (Figure 5A). This increase occurred at a very low degree, reaching 1.2~1.3 fold levels of the control cells at most, but it was sustained for days (Figure 5B) and quite different from the rapid and transient rise of Ca^2+^ upon mitogenic stimulation [40]. Additionally, this increase did not affect the physiology of the treated cells (in our previous studies, the growth rates of fibroblasts and keratinocytes were not altered upon treatment of 5 mM NAM [16,18]). These findings suggest that the increase of cytosolic Ca^2+^ is not mediated by typical mitogenic signaling and is likely to have arisen through slow release from either intracellular stores or by an influx across the plasma membrane. This change of cytosolic Ca^2+^ was reproduced by activation of SIRT1 upon SRT1720 treatment (Figure 5C,D). Further, NAM-induced increase in cytosolic Ca^2+^ was attenuated in cells transfected with siSIRT1 RNA. These together strongly suggest that the increase in cytosolic Ca^2+^ is an event downstream of SIRT1 activation.

### 3.6. Drp1 Activation Is Necessary for the Increase in Cytosolic Ca^2+^

**Figure 6:**
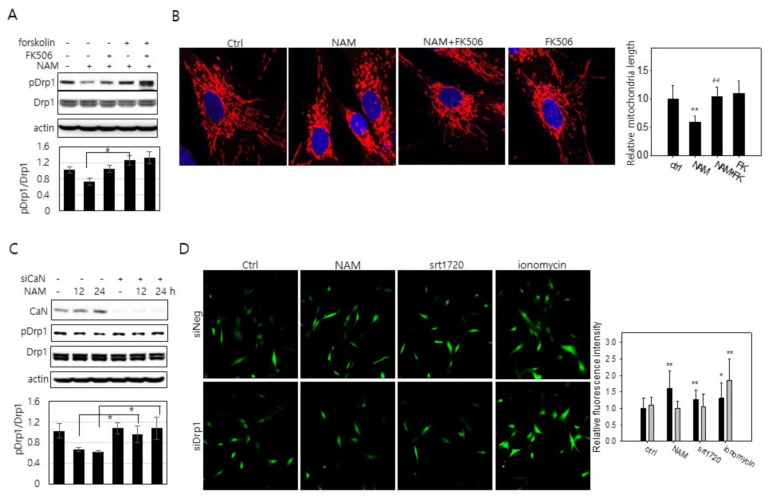
NAM-induced mitochondrial fragmentation is in part driven by calcineurin activation through an increase of Ca^2+^ that is dependent on Drp1. (**A**,**B**) Human fibroblasts cultivated with 5 mM NAM for 24 h with 2 μM FK506 for 1 h or 20 μM forskolin for 1 h prior to harvest were lysed and applied to immunoblotting analysis with antibodies against phosphor-Drp1 or Drp1 (**A**). Mitochondria were immuno-stained with Tom20 antibody and visualized by confocal microscopy (**B**). Nuclei were counter-stained with DAPI (blue). Length of mitochondria in at least 20 cells was measured and averaged, and mean values normalized by that of control cells were plotted in the bar graph. ## indicates a significant difference (*p* < 0.01) between the measured means of the cells untreated and treated with forskolin. (**C**) Cells transfected with siNeg or siCalcineurin RNA were incubated with 5 mM NAM for the indicated period, and then applied to immunoblotting analysis with antibodies against calcineurin, phosphor-Drp1, and Drp1. In (**A**,**C**), protein bands were quantitated, and the relative values of pDrp1 over Drp1 were normalized by those of control cells. Mean values of three different experiments were plotted and presented in bar graphs below. (*p* < 0.05) between NAM’s effects in the presence and absence of forskolin (**A**) or calcineurin (**C**). (**D**) Cells transfected with siNeg RNA or siDrp1 RNA were cultured in the presence of 5 mM NAM for 48 h, 1 μM SRT1720 for 24 h, or 2μM ionomycin for 5 min. After treatment, cytosolic calcium was stained with Fluo-4 and visualized in situ with fluorescence microscopy. The fluorescence intensities of at least 40 cells in multiple confocal images were quantified by using ImageJ program, and average values were plotted in the bar graph at right after normalization by those of control cells. Black bars and grey bars represent the values of the cells treated with control siRNA and siDrp1 RNA, respectively (*, *p* < 0.05 and **, *p* < 0.01 vs. control).

**Figure 7:**
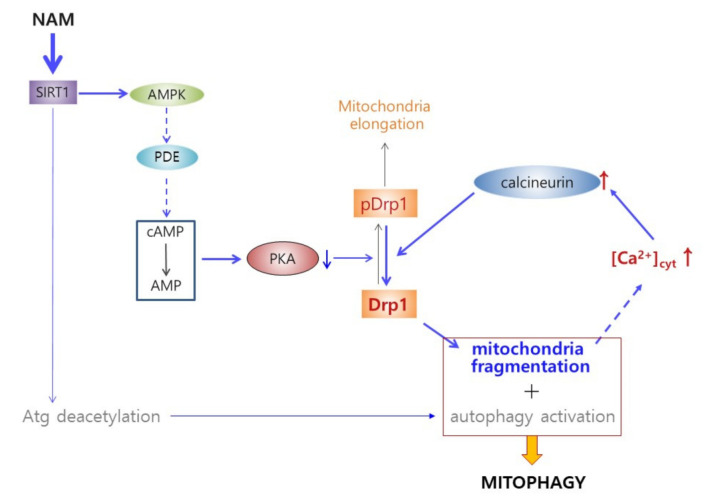
Hypothetical pathways for induction of mitochondrial fragmentation by NAM or SIRT1 activation. NAM treatment activates SIRT1 and increases AMPK activity. Activated AMPK can activate PDE4B causing conversion of cAMP to AMP, which downregulates PKA activity. As a consequence, Drp1 phosphorylation level decreases, and thereby mitochondria become fragmented. This mitochondrial fragmentation may lead to elevation of cytosolic Ca^2+^, which drives calcineurin activation and Drp1 dephosphorylation. This cycle of Drp1 hypo-phosphorylation—mitochondrial fragmentation—Ca^2+^ elevation—Drp1 dephosphorylation could accelerate mitochondrial fragmentation as well as sustain this state of mitochondria. This pathway of mitochondrial fragmentation (blue arrows) is also mobilized by direct activation of SIRT1 and AMPK. In coordination with autophagy activation, this SIRT1-mediated mitochondrial fragmentation would facilitate mitophagy. Dash arrows indicate the parts of the pathway that are not demonstrated clearly in this study.

Whether the increase in cytosolic Ca^2+^ plays a role in calcineurin-mediated Drp1 hypo-phosphorylation and mitochondrial fragmentation was investigated. Treatment of tacrolimus (FK506), a calcineurin inhibitor [41], to cells cultured in the presence of NAM for 12 h attenuated Drp1 hypo-phosphorylation (Figure 6A, lane 3), as did forskolin treatment (lane 4). FK506 treatment also attenuated NAM treatment-induced reduction in mitochondria length in NAM-treated cells (Figure 6B). Furthermore, the level of phosphor-Drp1 was not lowered when calcineurin expression was knocked down (Figure 6C). These results together suggest that calcineurin is indeed activated in NAM-treated cells and contributes to the decrease in the Drp1 phosphorylation level. Meanwhile, when Drp1 expression was knocked down, treatment of NAM or SRT1720 failed to elevate cytosolic Ca^2+^ (Figure 6D; see grey bars in the graph at right), indicating that the increase in cytosolic Ca^2+^ by the treatment of NAM or SIRT1 activation is a consequence of Drp1 activation. This suggests that the elevation of cytosolic Ca^2+^ is likely an outcome of mitochondrial fragmentation. Taken together, these findings indicate the presence of a cycle of Drp1 activation and cytosolic Ca^2+^ increase that further facilitates mitochondrial fission (Figure 7).

## 4. Discussion

Our study demonstrates that Drp1 activation occurs and is at least partially responsible for mitochondria fragmentation and enhanced mitophagy in NAM-treated cells. This study also reveals the presence of a pathway to Drp1 activation. In this pathway, SIRT1 activation driven by an increase of [NAD^+^] induces AMPK activation, which then drives Drp1 activation through induction of a decrease in [cAMP] and PKA activity. In this pathway, an additional factor likely plays a role. Mitochondria fragmentation itself induces an increase of cytosolic [Ca^2+^] and subsequently calcineurin activity and drives Drp1 dephosphorylation (Figure 7). Through these dual and reciprocal events, mitochondria fragmentation may be accelerated and also sustained in NAM-treated cells. 

Our study suggests the involvement of SIRT1 in Drp1 activation and thereby mitochondria fragmentation. SIRT1 activates mitophagy [19], in which mitochondria fission occurs in coordination with autophagy initiation. SIRT1 activates autophagy through deacetylating and activating key components of autophagy gene proteins [20] or FOXO3a, which induces the expression of autophagy promoting factor Bnip3 [42]. Both our own and other studies have shown that SIRT1 also induces mitochondria fission; therefore, it appears to function as a key player in mitophagy execution. In a previous study [43], an increase in the cellular NAD^+^/NADH level, which accompanied a decrease of ATP production, activated SIRT1 and AMPK and drove mitochondria fragmentation. This previous study also proposed that mitochondrial fragmentation is induced by SIRT1-mediated deacetylation of cortactin, which induces actin polymerization, rather than by modulating Drp1 activity; however, the effect of SIRT1 activation as an outcome of inhibition of IP3 receptor, which blocks Ca^2+^ transfer to mitochondria [44,45], was observed, so that the possible involvement of other effects of Ca^2+^-transfer block cannot be ruled out. In addition, this result was observed in HeLa, cancer cells, whose energy and Ca^2+^ metabolisms may be different from those of normal fibroblasts; therefore, it may not be relevant to normal cells. Nonetheless, along with Drp1-independent mitochondrial fragmentation, our results propose physiological complexity in the regulation of mitochondrial dynamics.

The mechanism underlying the decrease in the cAMP level in NAM-treated cells is not confirmed in this study. However, AMPK activation has been shown to decrease PKA activity and downstream PKA target phosphorylation through activating PDE4B [37]. AMPK-induced PDE4B activation might have occurred and contributed to the decrease in the cAMP level in NAM-treated cells. This decrease in cAMP is expected to cause changes in the physiology of cells, especially those that are sensitive to signaling for mitogenic stimulation, and may be related to various unanswered positive effects of NAM on neurons and brain cells [46].

The mechanism by which cytosolic Ca^2+^ increased in cells with fragmental mitochondria is not clear either. It has been noted that fragmental mitochondria that fail to reach the plasma membrane rather slowly accommodate incoming Ca^2+^ within their tubular space and therefore leave more Ca^2+^ in the cytosol [47]. If sustained, this may result in a substantial increase in cytosolic Ca^2+^. If this is the case, the possible presence of a self-stimulatory cycle of mitochondrial fragmentation is suggested. In this cycle, mitochondrial fragmentation occurs through two step-activation of Drp1, where initial activation induces first phase mitochondrial fragmentation, and this is followed by an increase of cytosolic Ca^2+^, which causes further Drp1 activation through calcineurin activity and a sustained maintenance of fragmental mitochondria (Figure 7). Although the method by which NAM treatment induces an increase of cytosolic Ca^2+^ was not clearly determined in the current study, one model, albeit a presumptive one, may be suggested. The mitochondrial network interacts with the plasma membrane at locations near Ca^2+^ release or influx channels and modulates ER store-operated Ca^+2^ entry [48]. Additionally, mitochondria–plasma membrane interaction facilitates rapid uptake of flowed-in Ca^2+^ ions [47]. Mitochondrial fragmentation induced by Fis1-overexpression causes detachment of the mitochondrial network from the plasma membrane and reduced Ca^2+^ uptake across plasma membrane channels. This model predicts that, in cells with fragmented mitochondria, Ca^2+^ ions entering through influx channels are taken up slowly by mitochondria with more of them accumulating in the cytosol, as appeared in the NAM-treated cells. Based on this prediction, calcineurin activation in NAM-treated cells is not caused directly through activation of unknown Ca^2+^ influx, but rather it is a consequence of mitochondrial fragmentation that is driven by initial Drp1 activation. Calcineurin activation can in turn cause further Drp1 activation through direct dephosphorylation, and thereby would cause continuous mitochondrial fragmentation. NAM, or activation of SIRT1 or AMPK, may also break Ca^2+^-homeostasis by altering Ca^+2^ flux through intracellular reservoirs, which may give rise to a modest elevation of Ca^2+^. A decrease in mitochondrial content upon NAM treatment may be another candidate mechanism for an increase in cytosolic Ca^2+^, but the slow kinetics in its change does not fit well with the rapid increase of cytosolic Ca^2+^.

This phenomenon of an increase in cytosolic Ca^2+^ upon NAM administration deserves further investigation. The small but sustained increase of cytosolic Ca^2+^ may have an impact on various cellular metabolisms and signaling. A change in the cytosolic Ca^2+^ level would modulate activities of mitochondrial enzymes and therefore alter energy metabolism [49,50]. NAM treatment at the dose used in this study, 5 mM, does not alter the proliferation and viability of the tested cells [16,18,19]. However, an increase in the cytosolic Ca^2+^ level would affect the activities of Ca^2+^-dependent enzymes such as calcineurin, as observed in this study. This effect may play an important role in cells such as neurons, whose activity and function are sensitively modulated by Ca^2+^ signaling; therefore, the results of this study may shed light on the neuro-protective effects of NAM against various neuronal diseases such as Alzheimer’s disease and cognitive decline [51] and motor deficits associated with Huntington’s disease phenotype [52]. NAM treatment also improves sensory and motor neurological behavior [53] and recovery from brain injury [54] and also ameliorates depression and psychological disorders [55,56]. This warrants investigation on NAM-induced Ca^2+^ mobilization on these neurological conditions as well as other degenerative diseases associated with aging.

## Data Availability

All relevant data are included within this article.

## Supplementary Materials

The following are available online at https://www.mdpi.com/2073-4409/10/3/612/s1: Figure S1: Changes in mitochondrial content and morphology and possible involvement of Drp1 but not Fis1 or Mfn1. Figure S2: Figure S2: Drp1 at S616 is not affected by NAM treatment. Figure S3: Involvement of SIRT1 activation in NAM-induced mitochondrial fragmentation and Drp1 activation. Figure S4: Effect of SIRT1 inhibition on phosphorylation of Drp1. Figure S5: Effect of AMPK knockdown on Drp1 phosphorylation and mitochondria length. Figure S6: Effect of IBMX and foskolin on NAM-induced change in the level of cAMP and Drp1 phosphorylation.
