# Supplementary figures and images for: Nicotinamide Treatment Facilitates Mitochondrial Fission through Drp1 Activation Mediated by SIRT1-Induced Changes in Cellular Levels of cAMP and Ca2+
Source: Cells. 2021 Mar 10;10(3):612. doi: 10.3390/cells10030612 (PMC7999186; doi:10.3390/cells10030612)

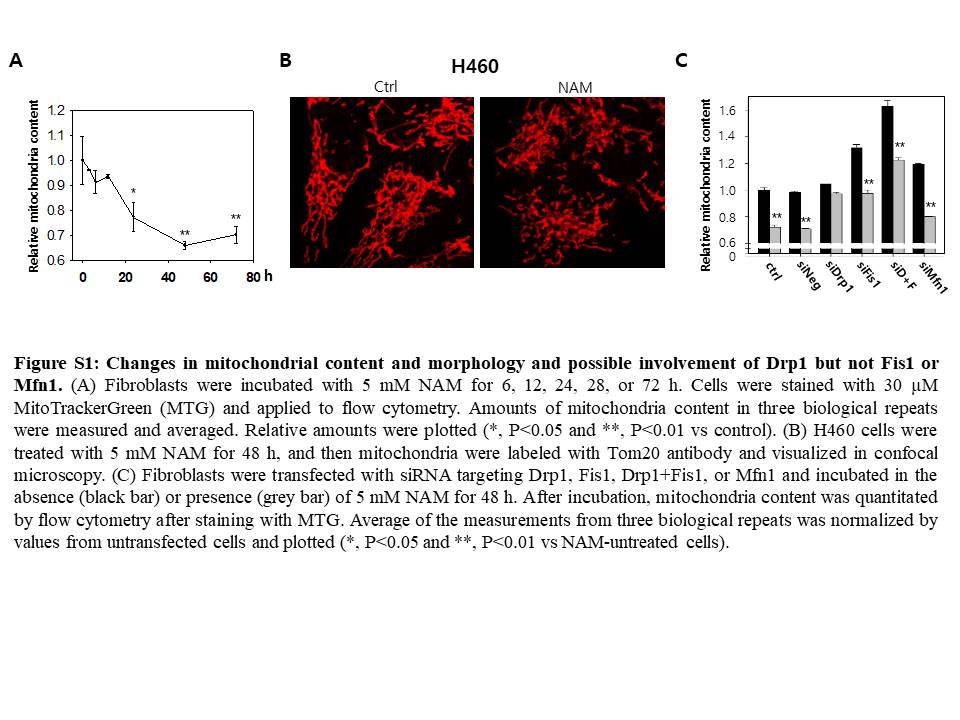

Supplement: Supplementary file 1 [file cells-10-00612-s001.zip › fig S1.JPG]

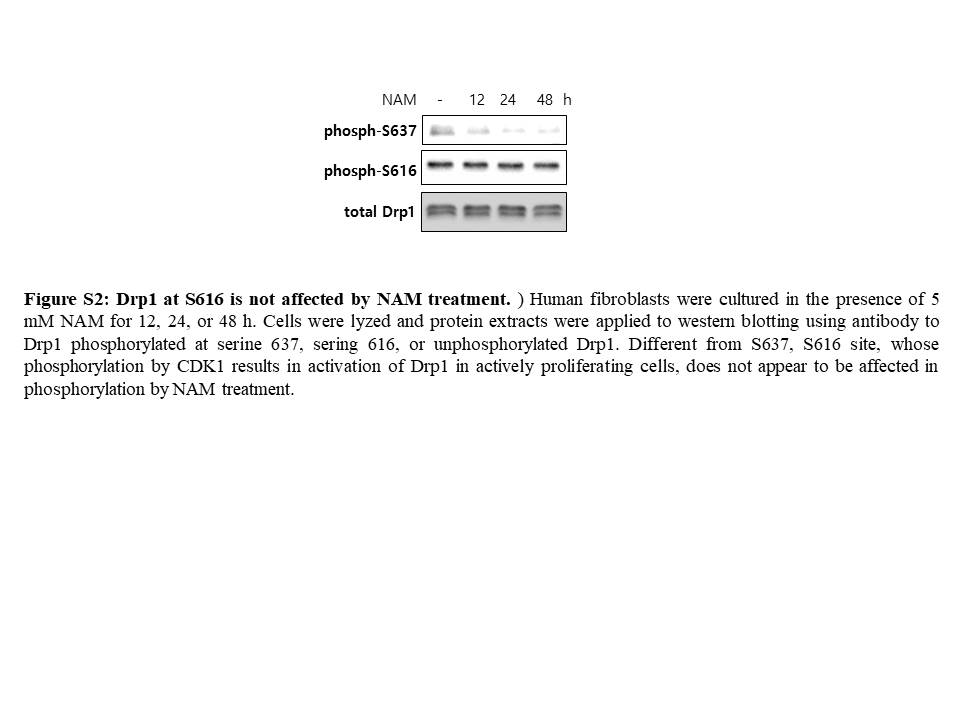

Supplement: Supplementary file 1 [file cells-10-00612-s001.zip › fig S2.JPG]

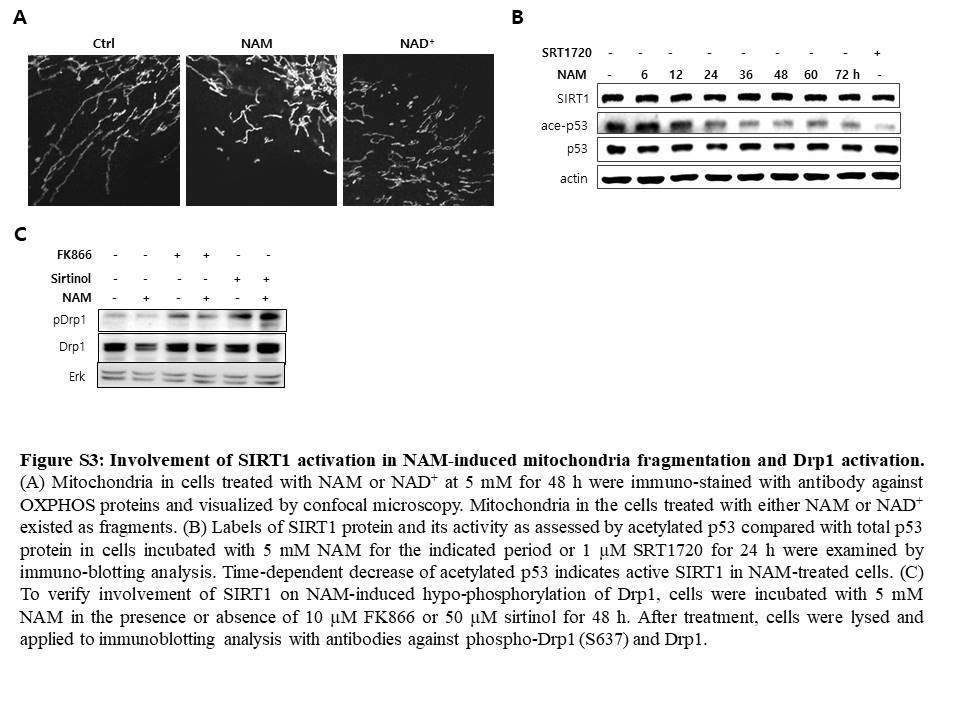

Supplement: Supplementary file 1 [file cells-10-00612-s001.zip › fig S3.JPG]

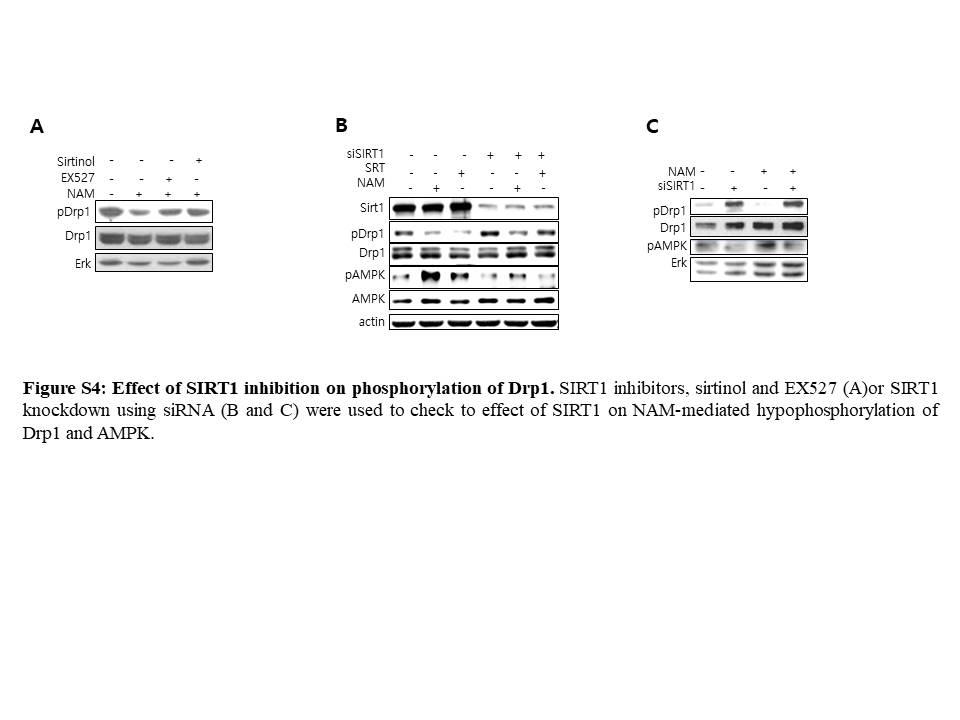

Supplement: Supplementary file 1 [file cells-10-00612-s001.zip › fig S4.JPG]

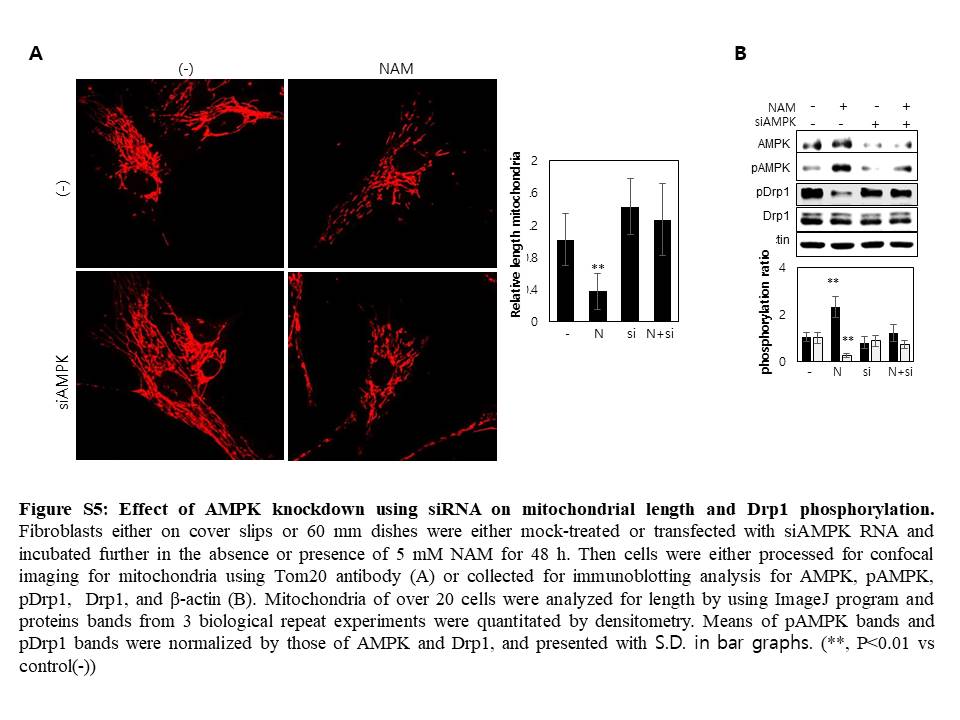

Supplement: Supplementary file 1 [file cells-10-00612-s001.zip › fig S5.JPG]

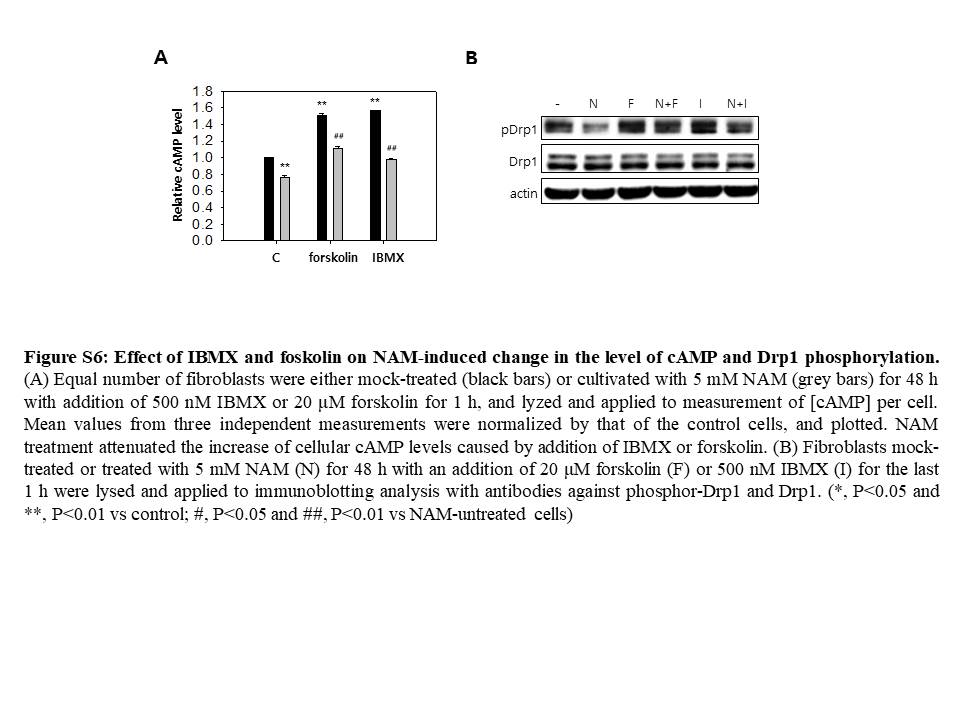

Supplement: Supplementary file 1 [file cells-10-00612-s001.zip › fig S6.JPG]
